# Corrigendum

**DOI:** 10.1002/ehf2.13215

**Published:** 2021-01-27

In the original published version of the paper of Giallauria *et al*.^1^ the data in *Table* *1*, under treatment (*n* = 36) column, 63.8 value was incorrect and has been changed to 94.4 in the online version. This change is marked in bold in the updated table below.

**Table 1.** Characteristics of the trials included

**Table:** 

	REDUCE FMR trial		TITAN trial		TITAN II trial
	Treatment (*n* = 73)	Control (*n* = 47)	Treatment (*n* = 36)	Control (*n* = 17)	Treatment (*n* = 36)
Gender (male) (%)	72.4	72.7	75	82	67
Mean age (years)	70.1 ± 9.7	69.1 ± 8.9	62.4 ± 12.7	62.6 ± 13.1	70.6 ± 8.5
Ischaemic aetiology, *n* (%)	59 (67.8)	21 (63.6)	24 (66.7)	10 (58.8)	21 (58)
Diabetes, *n* (%)	24 (27.6)	12 (36.4)	6 (16.7)	5 (29.4)	11 (31)
NYHA functional class (%)					
II	44.8	48.5	—	6	5.6
III	52.9	51.5	**94.4**	94	88.8
IV	2.3	—	5.5	—	5.6
Atrial fibrillation, *n* (%)	57 (58.6)	20 (60.6)	12 (33.3)	2 (11.8)	17 (47)
Device (ICD or PPM), *n* (%)	43 (49.4)	12 (36.4)	6 (16.7)	2 (11.8)	
6MWT distance (m)	306.4 ± 90.5	292.6 ± 91.5	302 ± 73.6	338 ± 83.4	294.1 ± 83
LVEF (%)	34 ± 9	37 ± 9	29 ± 7	27 ± 8	34 ± 10
LVEDV (mL)	187 ± 65.6	188.6 ± 75.7	208.5 ± 62.0	237.4 ± 96.7	174.4 ± 51.2
LVESV (mL)	127.4 ± 56.1	122.0 ± 59.8	151.7 ± 57.1	177.7 ± 91.9	119.8 ± 39.6
Mitral regurgitant volume (mL per beat)	40.4 ± 23.9	38.1 ± 24.0	34.5 ± 11.5	39.9 ± 13.2	34.4 ± 13.5
Mitral regurgitant grade (%)					
1+	28.7	32.3			
2+	39.1	25.8	19.4	11.8	28
3+	26.4	35.5	55.6	58.8	61
4+	5.7	6.5	25.0	29.4	11

There were also changes noted in *Figure* *2*. *Figure* *2* *D* title should read as ‘Changes in mitral regurgitation grade’, and *Figure* *2* *E* image was previously incorrect. The updated *Figure* *2* is shown below. Online version has been corrected too to reflect these changes.

**Figure 2:**
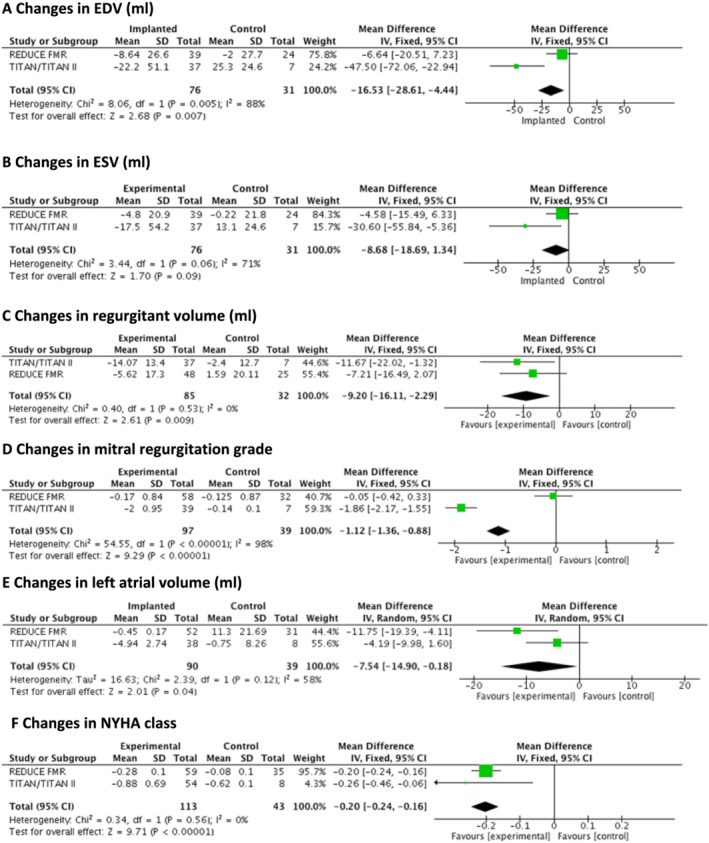
(A–F) Forest plots for changes in left ventricular remodelling, mitral regurgitation severity, left atrial volume, and New York Heart Association class at 12 month follow‐up. CI, confidence interval.
